# Decision making in human resource management: a systematic review of the applications of analytic hierarchy process

**DOI:** 10.3389/fpsyg.2024.1400772

**Published:** 2024-08-12

**Authors:** Reza Salehzadeh, Mehran Ziaeian

**Affiliations:** ^1^Department of Management, University of Isfahan, Isfahan, Iran; ^2^Department of Industrial Management, Yazd University, Yazd, Iran

**Keywords:** AHP, FAHP, ANP, human resource management, systematic review

## Abstract

The Analytic Hierarchy Process (AHP), Fuzzy Analytic Hierarchy Process (FAHP), and Analytic Network Process (ANP) methods are widely used for decision-making across various fields, and have shown success in numerous applications within human resource management (HRM). The purpose of this study is to present a systematic literature review on the applications of AHP, FAHP, and ANP in human resource management. The search process encompassed two main online databases, Scopus and Web of Science. This research covers a total of 180 application papers. To help readers extract quick and meaningful information, references are summarized in tabular format. The results showed that these methods have been applied in various domains of HRM such as performance management and appraisal, selecting human resources, talent attraction and retention, intellectual capital, workplace safety, reward management, e-HRM, green HRM, etc. To the best of our knowledge, no comprehensive research review has yet been conducted on the applications of AHP in HRM.

## 1 Introduction

Managerial decision making is the heart of all management functions (Wierenga, 2011), mainly because it affects the success or failure of an organization (Kozioł-Nadolna and Beyer, 2021). Many factors such as culture, ethics, lack of information, and external factors can affect the decision making process (Okonedo, 2018). However, there are times when a manager must make quick decisions about what to do and how to do it (Kharbanda and Stallworthy, 1990). Previous studies show that decision-making has fundamental importance in human resources management and organizational behavior (Salehzadeh, 2017; Gao et al., 2023; Luo and Zhu, 2023; Poveda, 2023). Human resource management is a holistic, integrated, and strategic approach to the employment, well-being, and development of the employees in the workplace (Armstrong, 2009). The main objective of human resource management is to ensure that the organization succeed through their employees (Salehzadeh et al., 2024). To compete with the rapidly changing environment, globalization and technological and scientific advances, organizations and governments must use a positive approach to attract, select, motivate, develop and retain the right employees (Shi and Lai, 2023). Therefore, the good decision-making in the field of human resource management is one of the main reasons that lead to increased organizational effectiveness (Noe et al., 2011).

Human resource managers play a key role in defining and implementing organizational strategies regarding people-related aspects such as talent management (Anlesinya et al., 2019), employee training and development (Solis, 2017), performance management (Armstrong, 2019), and compensation and benefits (Fahim, 2019). From this point of view, decisions made by HR managers have a direct impact on the competitiveness of companies (Walger et al., 2016). Wright et al. (2011) state that HR managers have become the second most important person in any organization after the CEO. As a result, the head of HR is under increasing internal and external pressure to deliver results. As employees are increasingly recognized as a source of competitive advantage (Davis, 2017), effective decision-making by human resource managers becomes even more critical. Most HRM practices take place in uncertain, vague and ambiguous conditions and are based on various methods and strategies with objective and subjective criteria. In addition, several factors with qualitative and quantitative nature influence HRM activities. In uncertain environments, the ability to engage in appropriate forms of decision-making is very important (Tabesh and Vera, 2020).

Decision-making methods and techniques can contribute and facilitate the decision-making process related to various HRM practices such as performance management (Sagawe et al., 2022; Nahoo and Kassam, 2023), selecting human resources (Ahirwal and Kumar, 2023; Tsai et al., 2023), talent attraction and retention (Luo and Zhu, 2023), etc. Among the decision-making methods, Analytic hierarchy process (AHP) technique is one of the most popular decision-making methods. AHP is a method for organizing and analyzing complex decisions based on math and psychology (Duan et al., 2022). Previous literature shows that many researchers have adopted AHP and fuzzy AHP methodology in various fields such as safety management system (Chan et al., 2004), selecting facility location (Chadawada et al., 2015), project selection (Parvaneh and El-Sayegh, 2016), e-government (Gupta et al., 2017), ranking halal parks (Islam and Madkouri, 2018), risk assessment (Lyu et al., 2020), and service quality (Bakir and Atalik, 2021). AHP has also been successfully used in various fields of human resource management (Peregrin and Jablonsky, 2021) such as selecting employees (Wu and Fang, 2011), human capital management (Tavakoli et al., 2016), green HRM (Goel et al., 2022) and employee performance (Gao et al., 2023). This method has been chosen for its versatility and high efficiency in solving different types of decision-making problems (Gupta et al., 2017; Peregrin and Jablonsky, 2021).

Although the AHP has found application in various human resource management areas, a comprehensive review of these applications remains absent in the current research landscape. This lack of a critical analysis represents a significant gap in our understanding of how AHP can be leveraged for effective HRM decision-making. This research offers several significant contributions. It will be the first comprehensive review of AHP applications in HRM. Moreover, it will assess and map the evidence of key features, research topics, and methodological decisions based on previous studies. Furthermore, it will delve into the application of AHP in emerging HRM areas like e-HRM and Green HRM. By providing a critical analysis of existing research, this article advances our understanding of how AHP can be used to improve decision-making in HRM. The results of this research have implications for several groups. The research will provide *HR professionals* with a comprehensive understanding of how AHP can be used to make better decisions in various HRM areas. This research will identify potential research gaps for *HRM researchers* and suggest future directions for studying AHP applications in HRM. In addition, by improving HR decision-making, this research can indirectly benefit *organizations* by leading to better workforce and organizational outcomes. The purpose of this research is to review the applications of AHP (and new improved and developed methods such as FAHP and ANP) in human resource management. In the next sections, we explain the concept of HRM and AHP. We then discuss about the applications of AHP, FAHP, and ANP in each of the HRM domains such as performance appraisal, selecting human resources, intellectual capital, workplace safety, reward management, e-HRM, etc. This is followed by the analysis of the findings, and the conclusion and discussion sections.

## 2 Theoretical backgrounds

### 2.1 Human resource management

In recent years, organizations have increasingly recognized the crucial role of human resources, placing it on par with critical areas like finance and marketing. This shift reflects a growing understanding that a skilled and motivated workforce is essential for achieving organizational goals (Safari et al., 2015). Deadrick and Stone (2014) argue that human resource management was probably the earliest evolved management function, predating others like finance, accounting, and marketing. According to Adla et al. (2020) there are different approaches on HRM in the literature. In the first approach, HRM practices are generally informal and leader-oriented (Lai et al., 2016). Paradoxically, in the second perspective HRM is more relational which results in better relationships between employees and managers (Psychogios et al., 2016). Many studies have been conducted in the history of human resource management research and practice (DeNisi et al., 2014). Based on Deadrick and Stone (2014) the evolution of HRM can be divided into four periods: (1) “Early beginnings” (1400s–1700s): this era saw the rise of division of labor and rudimentary management of human resources, often by tribal leaders. The industrial revolution (late 18th century) transformed work from manual to machine-based, creating a need for large-scale workforce management; (2) “Personnel” (1800s): the 1800s saw the emergence of “welfare-to-work” systems aimed at improving working conditions and attracting workers. These practices eventually evolved into modern employee benefits. Around the same time, “personnel managers” were introduced to handle hiring, firing, and other basic HR tasks; (3) “Human relations” (1900s–1970s): this era witnessed the rise of unions and scientific management approaches. The focus shifted to efficiency and productivity, often at the expense of worker well-being. The human relations movement emerged in response, emphasizing the social aspects of work and employee needs; and (4) “Strategic HRM” (1980s to present): the recognition of human resources as a strategic asset led to the development of “strategic HRM.” This approach emphasizes trust-building, and aligning HR practices with organizational goals.

Literature review shows that different definitions of human resource management have been suggested to date. Human resource management is a process of managing people through practices like recruitment and personnel selection, performance appraisal, reward systems, training and development (Lazarevic, 2001). In human resource management, management systems are designed to efficiently and effectively utilize human talent to achieve organizational goals (Mathis and Jackson, 2008). And human resource management consists of the systems, practices, and policies that affect the attitudes, behaviors, and performance of employees (Noe et al., 2011). Traditionally, human resource management was seen as a necessary cost, rather than as a source of value for organizations. However, many studies have shown that HRM practices can be valuable. Human resource practices can contribute to the strategic goals and performance of the organization (Truss, 2001; Wright et al., 2001; Boselie et al., 2005; Combs et al., 2006; Buller and McEvoy, 2012; Ridder et al., 2012; Sabiu et al., 2019). Effective decision-making is a cornerstone of successful human resource management, as evidenced by prior research (Luo and Zhu, 2023; Poveda, 2023). Decisions about hiring, training, and evaluating the employee performance directly influence employees’ motivation and have positive organizational outcomes (Vivares-Vergara et al., 2016; Katou, 2017). Therefore, it becomes clear why studying and refining HR decision-making processes is crucial. By continuously analyzing and improving the way managers make choices about their employees, they can build a more successful organization for the future.

### 2.2 Analytic hierarchy process

There are two main categories of Multiple Criteria Decision Making (MCDM) methods: Multiple Objective Decision Making (MODM) and Multiple Attribute Decision Making (MADM; He et al., 2016). MODM tackles continuous decision problems with potentially endless solutions or criteria. In contrast, MADM deals with discrete problems where the number of alternatives and criteria is finite (Cordoba Bueno, 2004). Analytic hierarchy process is one of the most common MADM methods (Shahin and Salehzadeh, 2021; Gao et al., 2023; Tavana et al., 2023) which is often used to make decisions in situations where there are multiple criteria/factors (Saaty, 1980). Analytic hierarchy process (AHP) is a structured method for making complex decisions and has been extensively studied and used for alternative ranking, prioritization, and selection (Tavana et al., 2023).

The AHP methodology is a linear MCDM assessment to provide weights and ranks compared to other MCDM methods such as DEMATEL, VIKOR, DEA, TOPSIS and ANP; and also different optimization and search techniques like Bayesian methods, Genetic algorithms, Multiobjective programming, etc. (Shi and Lai, 2023). The AHP methodology divides the multi-criteria decision-making problem into a hierarchy with at least three levels: goal (objective), criteria, and decision alternatives (Saaty, 1980). The AHP creates a hierarchical model of these three levels, evaluates the priorities of the criteria, compares the decision alternatives for each criterion, and finally determines the rank of these alternatives (Gupta et al., 2017). Given the critical role of decision-making in human resource management, quantitative techniques like the analytic hierarchy process offer valuable support. AHP’s versatility and efficiency in tackling various decision-making problems (Gupta et al., 2017; Peregrin and Jablonsky, 2021) make it a popular choice in the HRM domain (Wu and Fang, 2011; Peregrin and Jablonsky, 2021; Gao et al., 2023).

## 3 Methodology

**Figure 1:**
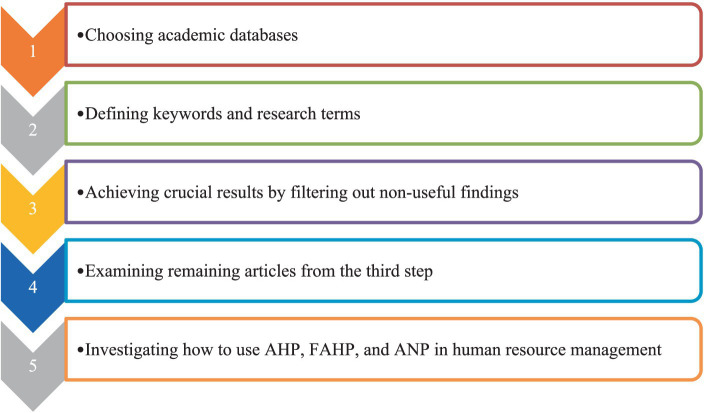
Research process steps.

In this research a systematic literature review method was used. This technique is a validated research method because it gives researchers confidence in understanding key concepts in the literature (Tranfield et al., 2003). Before conducting the systematic review and identifying keywords, we performed an exploratory search in valid databases to obtain sufficient information about the main concepts and keywords of the studies. Figure 1 shows the stages of the research, which includes several successive phases.

**Figure 2:**
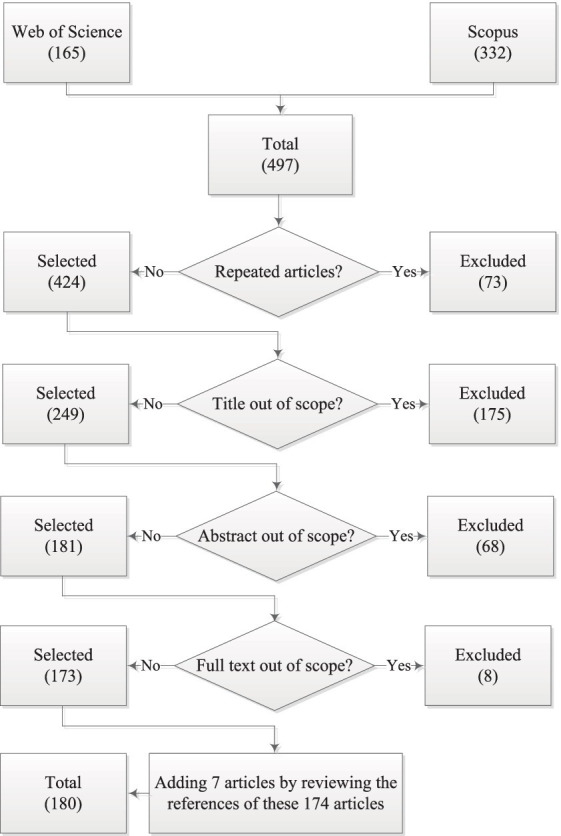
PRISMA flowchart of articles selection.

In the first step, we identified the primary academic research databases. Accordingly, two databases including Scopus and Web of Science were adopted. In the next step, we analyzed the studies extracted from these databases to select various search keywords such as (“Human resource management” OR “Human resource” OR “HRM” OR “HRM practices” OR “Employee selection” OR “Performance appraisal” OR “Talent management” OR “Succession planning”) AND (“Analytic Network Process” OR “Fuzzy Analytic Network Process” OR “Analytic Hierarchy Process” OR “Fuzzy Analytic Hierarchy Process”). Following the steps shown in Figure 2, we initially found 563 and 170 results in the Scopus and Web of Science databases, respectively. To refine the search and focus on more relevant articles, we limited our results to include only full-text, English-language articles published in academic journals. Additionally, we restricted the publication date to articles published up to 2023. After applying these filters, the initial results were reduced to 332 articles from Scopus and 165 articles from Web of Science (497 articles total). Out of 497 selected articles, 73 duplicate articles were removed using Mendeley software, leaving 424 articles. After reviewing the titles of these articles, 175 were considered irrelevant and excluded, leaving 249 articles. Subsequently, the abstracts of these articles were reviewed and 68 were excluded, leaving 181 articles. Then, after reading the full text of these 181 articles, 8 were excluded, leaving 173 relevant articles. In addition, by reviewing the references of these 173 articles, 7 articles were added to the selected articles. Therefore, at the end of this stage, 180 studies have been considered for final analysis.

## 4 Findings

### 4.1 Bibliometric analysis and results

**Figure 3:**
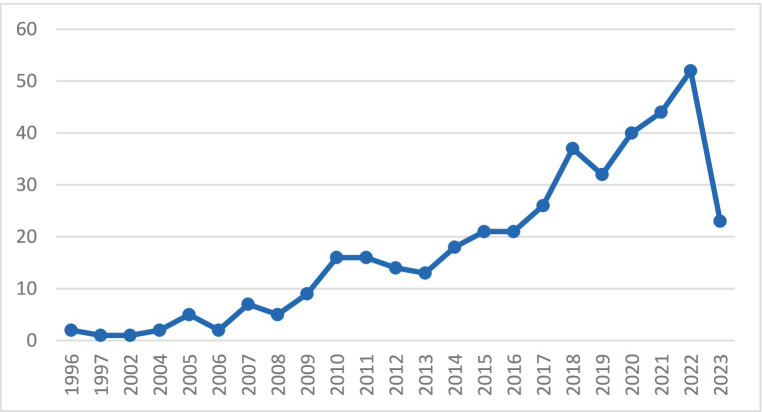
The trend of the number of conducted studies.

The trend of the number of studies conducted in different years based on 424 selected articles from the Scopus and Web of Science databases is shown in Figure 3.

**Figure 4:**
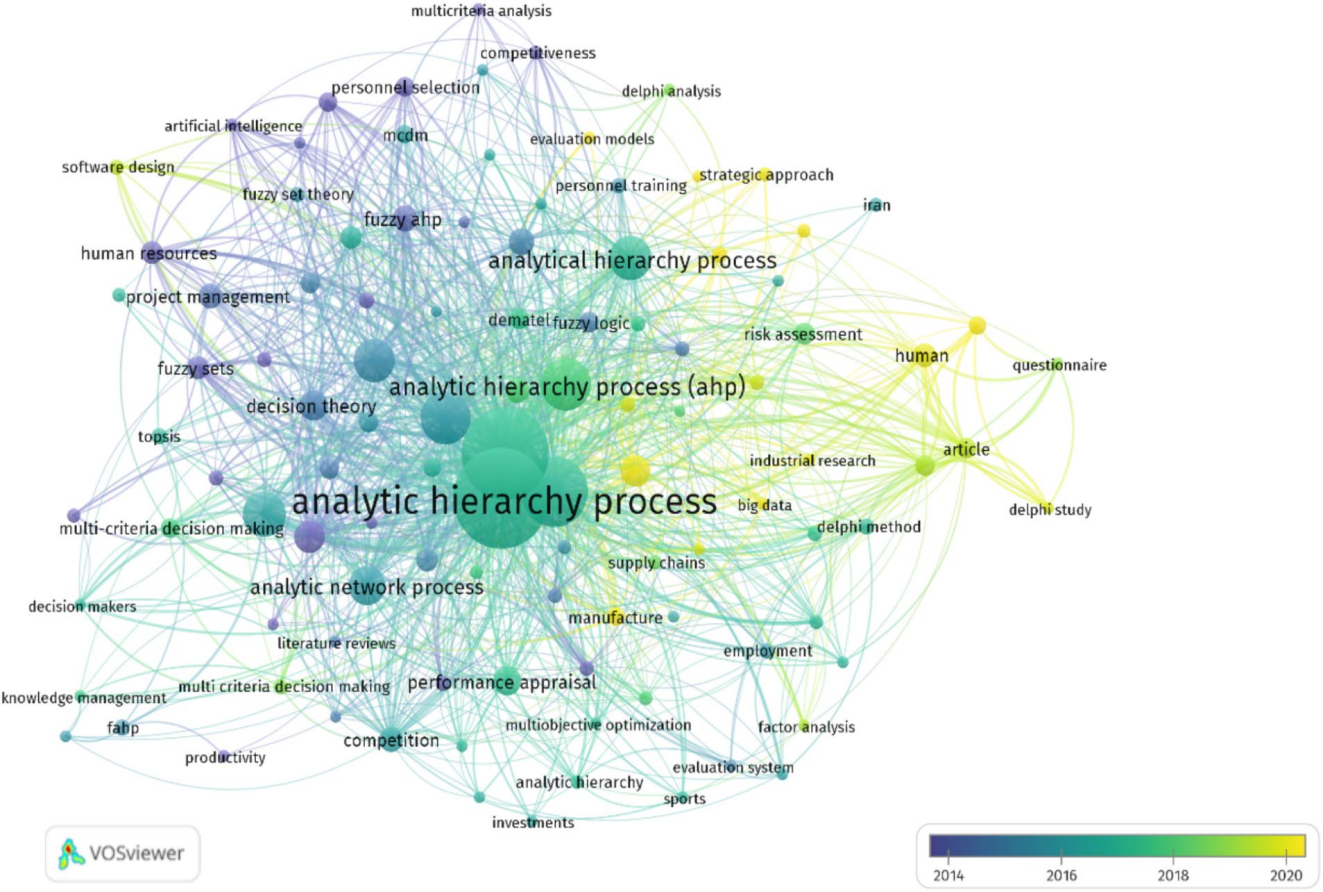
The relationships among the main keywords.

As shown in Figure 3, the trend of the conducted studies is generally upward. Since 2019, a significant number of studies have been conducted, showing the importance of two approaches, AHP and ANP. Figure 4 shows the words used in the 424 selected articles and how they are related to other words using the VOSviewer software.

**Figure 5:**
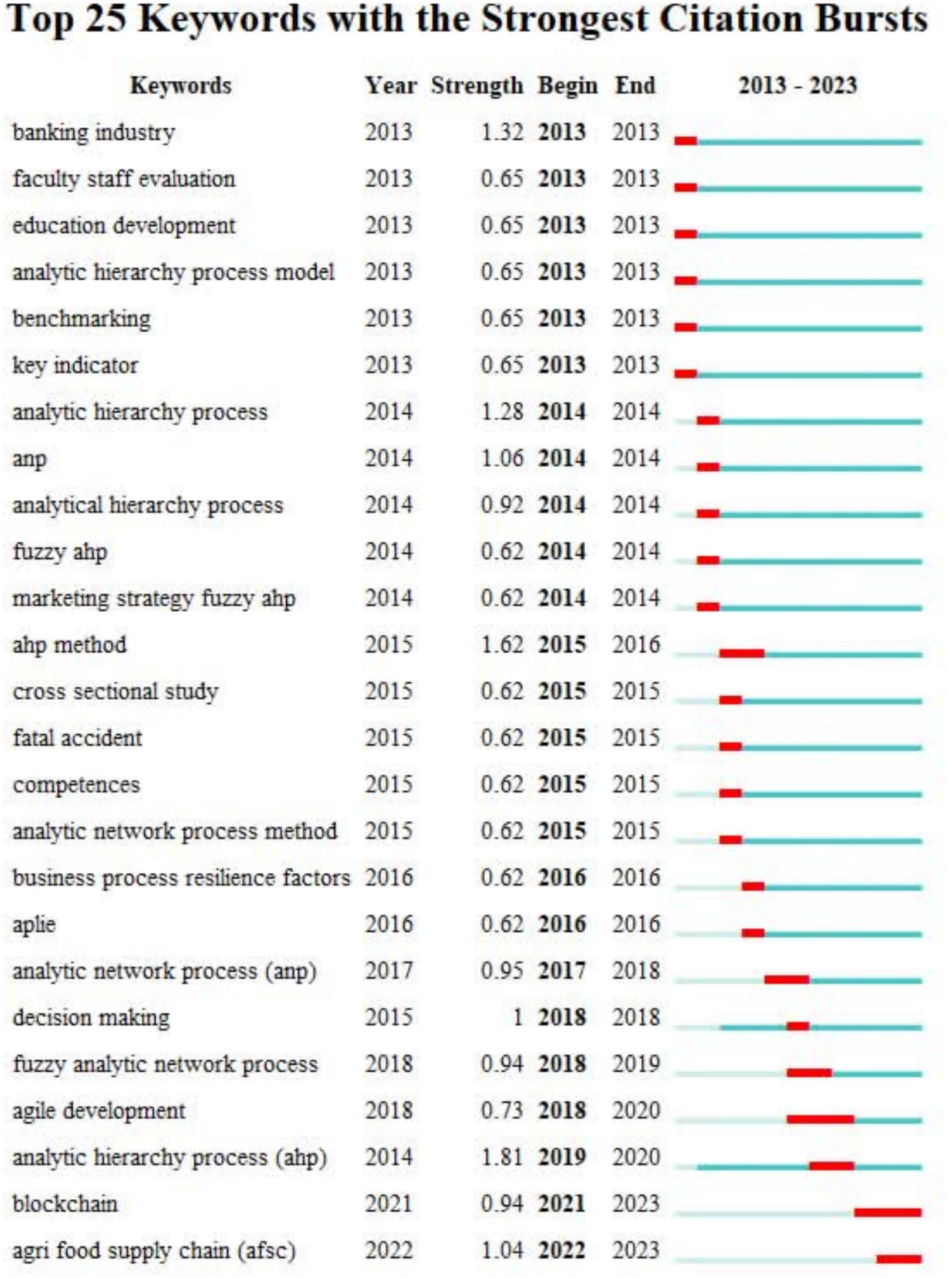
Keywords with the strongest citation bursts.

Examining the keyword co-occurrence network, as visualized in Figure 4, is a valuable technique for uncovering the central themes within a body of literature. This network allows us to understand the structure of a research field by mapping the relationships between keywords. Each node in the network represents a keyword, and the size of the node corresponds to the frequency of occurrence of that keyword within the analyzed text. As shown in Figure 4, keywords such as AHP, ANP, human resources, performance appraisal, multicriteria decision making, personnel training, evaluation models, decision theory and fuzzy logic are the most interrelated keywords. Figure 5 also shows the keywords with the strongest citation bursts using CiteSpace software.

**Figure 6:**
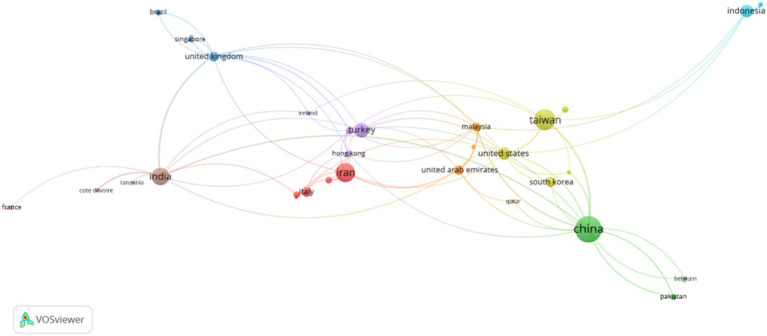
The relationships among countries.

The strongest citation bursts demonstrates the significance of specific keywords related to the topic across different time periods (Jiang et al., 2019). For example, as shown in Figure 5, in the field of application of AHP in HRM in 2013, keywords such as banking industry and employee evaluation were highly popular. Figure 6 shows the cooperation of different countries.

**Figure 7:**
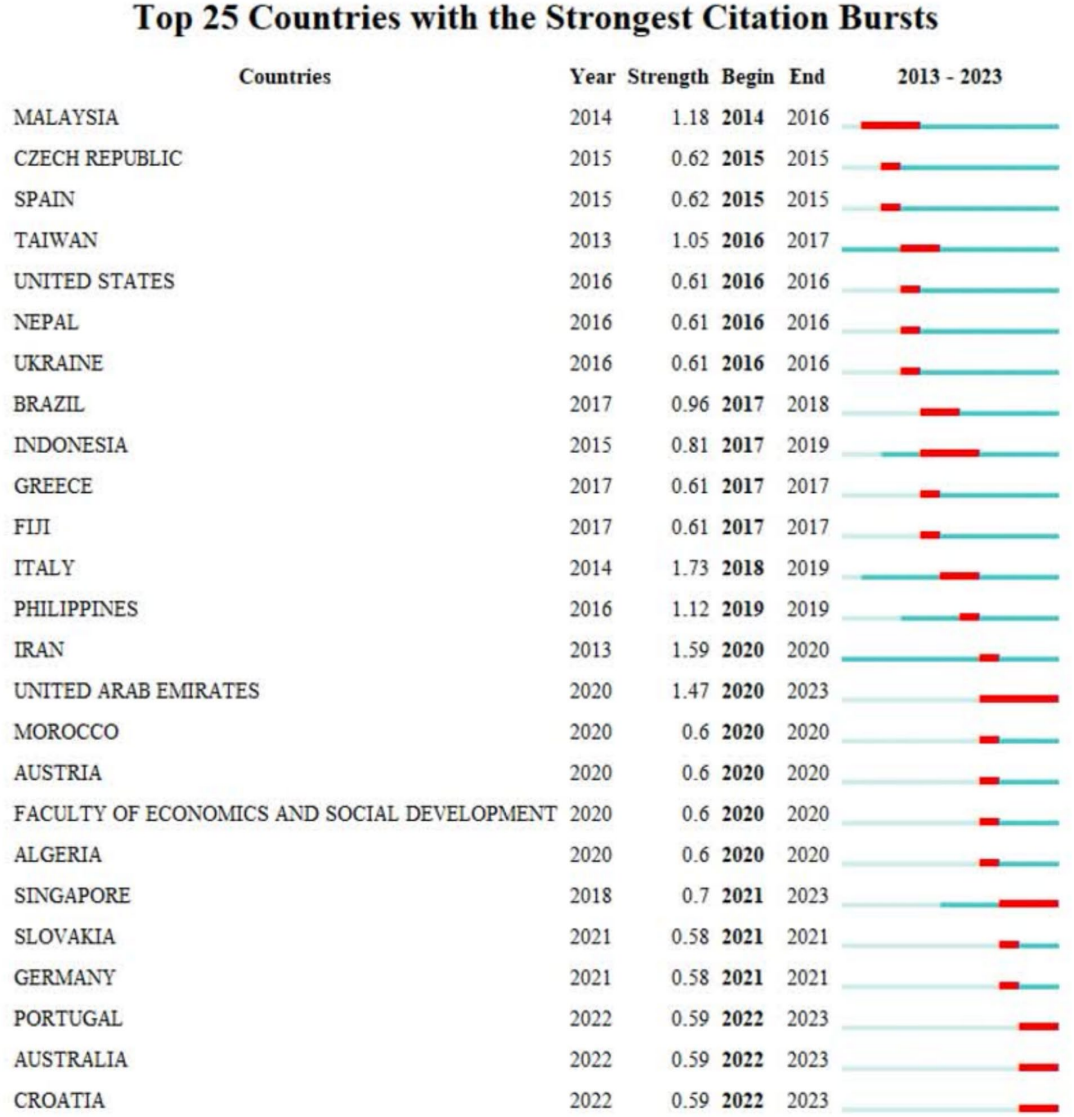
Countries with the strongest citation bursts.

As shown in Figure 6, Asian countries, including Iran, China, Malaysia, etc., had the most articles. Figure 7 also shows the countries with the strongest citation bursts. Countries with the strongest citation bursts reveal shifts in research focus across different regions over time within the field of human resource management. For instance, during the 2014–2016 period, articles published in Malaysia showed a particular emphasis on HRM as a whole, along with specific HR issues like employee selection, training, and performance. As Figure 7 illustrates, the majority of the strongest citations to published articles between 2014 and 2023 originated from Asian and European countries. This trend suggests a particular focus on HRM research and high-quality article publication in these two continents compared to others.

### 4.2 HRM domains and AHP

In this section, we specifically review the final 180 selected articles on the application of AHP in various HRM domains (see Table 1).

**Table 1:** HRM domains and performed studies.

HRM domains	Performed studies
Performance management and appraisal	Gao et al. (2023), Yiğit, 2023, Feng et al. (2023), Başaran et al. (2023), Astiti and Darmawan (2023), Gopi and Subramoniam, 2023, Nahoo and Kassam, 2023, Sagawe et al. (2022), Zare et al. (2022), Tang (2022), Yang (2022), Xia et al. (2022), Quezada et al. (2022), Kumar et al. (2022), Abbasi et al. (2022), Alshurideh et al. (2022), Yan and Chu (2021), Rezaie et al. (2021), Leilaee and Rezaeian (2021), Heravi et al. (2021), Oborenko et al. (2020), Mehrajunnisa and Jabeen (2020), Jafari et al. (2020), Boulagouas et al. (2020), Nurhayati (2019), Rezaeian et al. (2019), Longo et al. (2019), Liu et al. (2019), Chou et al. (2019), Celis and Pagatpatan (2019), Li (2018), Ponsiglione et al. (2018), Quaigrain and Issa (2018), Mirahmadi et al. (2018), Lidinska and Jablonsky (2018), Çelikbilek (2018), Beskese et al. (2018), Yu (2017), Tsai and Lin (2017), Sucahyo et al. (2017), Rahimnia et al. (2017), Qu et al. (2017), Jihui and Lei (2017), Irhamni et al. (2017), Ghassabi (2017), Chen et al. (2017, 2018), Kashi (2016), Ishizaka and Pereira (2016), Shafii et al. (2015), Manafi and Subramaniam (2015), Singh and Aggarwal (2014), Pan (2014), Sedaghat (2013), Oh et al. (2013), Do and Chen (2013), Dincer and Hacioglu (2013), Gomes and de Andrade (2012), Sepehrirad et al. (2012), Min-Peng et al. (2012), Manoharan et al. (2011), Hung and Jin (2011), Huang et al. (2011), Toloie-Eshlaghy and Peydaie (2011), Lin et al. (2010), Fang et al. (2010), Erensal et al. (2010), Hong-Lei et al. (2009), Adamus (2009), Lin and Meng (2009), Mian (2009), Chen and Lee (2007), Cheng and Li (2006), Islam and Mohd Rasad (2006), Albayrak and Erensal (2004), and Taylor et al. (1998)
Selecting human resources	Hashemkhani Zolfani and Antucheviciene (2012), Daneshvar Rouyendegh and Erman Erkan (2012), Rouyendegh and Erkan (2012), Mojahed et al. (2013), Katerina and Vaclav (2013), Varmazyar and Nouri (2014), Zarei and Wong (2014), Hadikurniawati and Wardoyo (2015), Lele (2015), Skrzypek and Dąbrowski (2015), Thakre et al. (2017), Abbasianjahromi et al. (2018), Gustilo and Escolar-Jimenez (2019), Vraňaková et al. (2019), Bhattacharya et al. (2020), Chuang et al. (2020), Chang (2020), Lin et al. (2020), He et al. (2021), Peregrin and Jablonsky (2021), Wang et al. (2022), Ahirwal and Kumar (2023), Tantranont and Sawatdeenarunat (2023), Tsai et al. (2023), Wu and Fang (2011), Shahhosseini and Sebt (2011), Hsiao et al. (2011), Ardabili (2011), Hor et al. (2010), Daǧdeviren (2010), Wu et al. (2009), Polychroniou and Giannikos (2009), Korkmaz et al. (2008), Gungor et al. (2008), Boran et al. (2008), Azar and Latifi (2008), Saaty et al. (2007), Gibney and Shang (2007), Momeni and Jahanbazi (2007), Chiang and Wang (2007), Golec and Kahya (2007), Shih et al. (2005), Huang et al. (2001, 2004), Lazarevic (2001), and Tavana et al. (1996)
Talent attraction and retention	Huang and Wu (2005), Vahdat and Farshid (2011), Mavi and Mavi (2014), Yang (2017), Lai and Ishizaka (2020), Yildiz et al. (2020a,b), and Luo and Zhu (2023)
Intellectual capital	Bozbura et al. (2007), Bozbura and Beskese (2007), Asonitis and Kostagiolas (2010), Abdullah et al. (2013), Wang and Hwang (2014), Tavakoli et al. (2016), Ghassabi (2018), Wang (2022), and Poveda (2023)
Workplace safety	Dagdeviren and Yuksel (2008), Melemez (2015), Zhang et al. (2019), Li et al. (2022, 2023), and Guan et al. (2023)
Reward management	Aksakal and Dağdeviren (2014) and Shi and Lai (2023)
e-HRM and digital technologies	Farsijani and Aref Nejad (2011), Lo et al. (2011), Faliagka et al. (2012), Saeedi Aghdam et al. (2014), Gupta et al. (2017, 2022), Priyadarshinee et al. (2017), Santoso et al. (2021), and Habib and Sajid (2022)
Green HRM	Gandhi et al. (2016), Thakur and Mangla (2019), D’Adamo (2022), Goel et al. (2022), Khatoon et al. (2022), Mehrajunnisa et al. (2022), Milošević et al. (2022), and Alavi and Aghakhani (2023)
Knowledge management	Castrogiovanni et al. (2016), AlShamsi and Ajmal (2018), Sani et al. (2019), and Muniz et al. (2022)
Other applications	Panazan et al. (2023), Joshi et al. (2017), Abdullah and Zulkifli (2015), Chou et al. (2012), Lin and Hsu (2010), Wu et al. (2010), Lin et al. (2009), Tseng and Lee (2009), and Kwak et al. (1997)

#### 4.2.1 Performance management and appraisal

Several studies have been conducted on the applications of AHP and FAHP in performance appraisal. For example, Gao et al. (2023) used AHP approach to improve psychological empowerment and employee performance. Feng et al. (2023) used the AHP method to determine strategic human resource management ability in the clinical departments of public hospitals. Sagawe et al. (2022) used the AHP and Electra approaches to identify the most important criteria related to talented and capable employees. Zare et al. (2022) used the AHP approach to evaluate human resource efficiency in the petrochemical industry. In this research, they have identified the most important human errors and factors affecting human performance, and ranked and prioritized them using the AHP method. Nurhayati (2019) evaluated the performance of employees using AHP approach. In this research, he identified criteria such as work attitude and personality as the most important criteria for evaluating employees’ performance. Kashi (2016) applied the AHP approach for ranking the individual competencies of senior managers in the automotive industry. Sepehrirad et al. (2012) developed a hybrid mathematical model based on the several tools such as fuzzy AHP, 360° performance appraisal, simple additive weighting (SAW), TOPSIS, mathematical model, and Delphi method for performance appraisal. Min-Peng et al. (2012) applied AHP and fuzzy comprehensive evaluation to assess the performance of engineering R&D staff. Manoharan et al. (2011) integrated several tools such as fuzzy analytic hierarchy process, Fuzzy Multi-Attribute Decision Making (FMADM), and Fuzzy Quality Function Deployment (FQFD) to evaluate employee performance. They organized the steps of their method as follows. Identification of main and sub-factors every 2 or 3 years through discussion with supervisors and using literature review; determining weight for main factors using fuzzy AHP; determining weight for sub-factors using fuzzy QFD; evaluating and ranking employees every 6 months or annually using fuzzy MADM; set target values for all sub-factors using fuzzy MADM; and performance improvement and skill development through training. Hong-Lei et al. (2009) established a system for evaluating the effectiveness of human resource management of commercial banks using the analytic hierarchy model. First, they identified the important indices and then used the analytic hierarchical model to create a pairwise comparison matrix and obtain the weight of each index. Finally, based on the obtained results, they created a system for evaluating the effectiveness of human resource management. Adamus (2009) proposed a new method for job evaluation. He used seven main criteria (cooperation, responsibilities, intellectual effort, physical and mental effort, wisdom, experience, and know-how and skill); and 38 sub-criteria and applied the AHP approach to determine the weights of each (sub) criteria. Islam and Mohd Rasad (2006) used AHP method for personnel performance appraisal. Albayrak and Erensal (2004) applied AHP to solve the human performance improvement problem. They used AHP for structuring and explaining the relationship between management style and human performance improvement. Taylor et al. (1998) applied AHP technique for employees’ performance evaluation.

#### 4.2.2 Selecting human resources

Many studies have been undertaken in the field of employee selection using AHP, FAHP, and ANP. Tsai et al. (2023), using decision-making techniques, investigated and ranked the key determinants of employee selection. Peregrin and Jablonsky (2021) used the AHP and ANP approaches to select appropriate employees in the recruitment process. Varmazyar and Nouri (2014) presented a FAHP approach for hiring the employees. They developed a computer-based program to evaluate and rank the candidates using the appropriate voting system. Mojahed et al. (2013) applied a mixed method of Electre-AHP for personnel selection. Based on Katerina and Vaclav (2013) many companies use the competency models in their human resource management systems. These models help human resource management department in recruitment and succession planning. They used AHP to determine and rank the core competencies of top managers. Daneshvar Rouyendegh and Erman Erkan (2012) applied FAHP for selection of academic staff. Wu and Fang (2011) combined the FAHP and the fuzzy Delphi methods to develop critical competences of professional managers. Shahhosseini and Sebt (2011) used fuzzy set theory for the selection of personnel. First, they classified human resources into four categories: laborer, technician, engineer, and project manager. Then, they developed the competency criteria model for each category. They performed decision making in two steps: FAHP to evaluate the competency criteria, and ANFIS to establish the competency IF-THEN rules of the fuzzy inference system. Finally, they used a hybrid learning algorithm for training the system. Hor et al. (2010) designed a leadership development program to decide how to select the leaders using ANP. Korkmaz et al. (2008) presented an analytic hierarchy process and two-sided matching-based DSS for military personnel assignment. Gungor et al. (2008) presented a fuzzy analytical hierarchy process for employee selection problem. This method considered both qualitative and quantitative criteria. The authors introduced a computer-based DSS to help managers in making better decisions under fuzzy circumstances. Boran et al. (2008) developed an ANP model for personnel selection. Azar and Latifi (2008) considered five criteria for selecting the human resource managers, include personal features, managerial skills, personal abilities, how to interact with superiors, and the acceptance and cooperation of employees. They used AHP method to rank these criteria and their sub-criteria. Saaty et al. (2007) used the combined AHP and Linear Programming (LP) models to optimize human resource allocation problems. Gibney and Shang (2007) applied AHP method to the dean selection. They used two criteria of leadership and resources as the main criteria. The leadership criterion included two sub-criteria: interpersonal/environmental skills, and vision. The resources criterion included two sub-criteria: internal and external focus. Momeni and Jahanbazi (2007) designed a fuzzy multiple criteria decision-making model for selecting the managers. After identifying the competency criteria, they used AHP method to determine the weight of main criteria. Then using fuzzy set methodology and TOPSIS technique, they ranked the management candidates. Chiang and Wang (2007) applied fuzzy AHP to evaluate the management competencies for middle managers. Shih et al. (2005) proposed a group decision support system (GDSS) involving various techniques such as AHP and TOPSIS for selecting the right person for the right job. Huang et al. (2004) used a combination of tools such as fuzzy neural network, SAW, and FAHP to build a new model to assess the managerial talent, and consequently to create a DSS in human resource selection. They used FAHP to allow decision-makers adjust weighted values and gain definitive results of each step’s scores. Lazarevic (2001) presented a fuzzy model for employee selection. This model consists of an analytic hierarchy process of three levels. The first level is the preliminary selection. The second level includes the selection process of a final applicant for a job position, and the third level includes the hiring the appropriate employee. Huang et al. (2001) adopted the fuzzy AHP to develop a model of managerial competences. Tavana et al. (1996) proposed a managerial selection framework. Their proposed group decision support system combined the AHP with the Delphi method to rate each candidate.

#### 4.2.3 Talent attraction and retention

Based on Luo and Zhu (2023), a good public service system and a standardized management platform are important factors that affect the concentration of top talents. They established a reasonable and scientific evaluation system of talent attraction using the AHP method. Lai and Ishizaka (2020) applied multi-criteria decision analysis methods into talent identification process. Yang (2017) used a fuzzy evaluation model of creative talents based on analytic hierarchy process. Mavi and Mavi (2014) presented a fuzzy analytic hierarchy process for ranking the attributes for talent pool membership in sport organizations. Huang and Wu (2005) applied a fuzzy analytic hierarchy process in the managerial talent assessment model. Yildiz et al. (2020a) used a hesitant fuzzy analytic hierarchy process for evaluation of positive experience of employees. Yildiz et al. (2020b) applied a spherical fuzzy analytic hierarchy process based approach to prioritize career management activities improving employee retention. Vahdat and Farshid (2011) used a fuzzy analytic hierarchy process to identify and prioritize factors affecting employee turnover.

#### 4.2.4 Intellectual capital

Asonitis and Kostagiolas (2010) presented a framework based on analytic hierarchy process, ISO 11620 international standards, and Delphi method for prioritizing intangible assets of intellectual capital. Bozbura et al. (2007) developed a methodology based on FAHP approach to improve the quality of ranking the human capital measurement indicators under fuzzy circumstances. First, they defined five main attributes include cultural relevance, knowledge management, strategic integration, talent, and leadership; their sub-attributes, and 20 indicators. Then, they ranked the measurement indicators of HC using FAHP method. Also, Abdullah et al. (2013) used AHP to rank the indicators of human capital. Poveda (2023) used AHP approach to assess the importance of human capital in meeting the goals and objectives of sustainable development. Tavakoli et al. (2016) used ANP and data envelopment analysis to rank organizational units and prioritize human capital drivers. Another sub-dimension of intellectual capital is organizational capital. Organizational capital is formalized knowledge in an organization, stored in manuals, databases, etc. (Youndt, 2000). Bozbura and Beskese (2007) presented a FAHP approach to improve the quality of ranking the organizational capital measurement indicators under uncertain circumstances. First, they defined three main attributes include the flexibility of the structure, investment in technology, and deployment of strategic values; their sub-attributes, and 10 indicators. Then, they ranked the measurement indicators of organizational capital using the FAHP method.

#### 4.2.5 Workplace safety

Since many factors affect the safety of work systems at the same time, a comprehensive approach is required to measure the work system safety. A fuzzy analytic hierarchy process approach allows for simultaneous and multi-criteria evaluation. For example, Dagdeviren and Yuksel (2008) developed a FAHP method to determine the level of faulty behavior risk in work systems. Their method was composed of three steps: (1) determining the factor and sub-factors for using in the model; (2) building a decision-making model using AHP; and (3) determining factor/sub-factor weights using FAHP.

#### 4.2.6 Reward management

Shi and Lai (2023) have used a fuzzy AHP approach to evaluate the incentive factors of high-tech talent agglomeration. They showed that for success in the use of advanced technologies, motivation of human resources is one of the most important individual factors. Aksakal and Dağdeviren (2014) using AHP and DEMATEL methods examined reward management as a framework based on four main criteria, including work environment, learning and development, benefits, and pay and three sub-criteria for each main criterion.

#### 4.2.7 e-HRM and digital technologies

Based on the literature review, several studies on the application of AHP in e-HRM were found. For example, Gupta et al. (2017) used AHP approach to rank and prioritize the factors influencing the acceptance of e-government by human resources. Saeedi Aghdam et al. (2014) applied AHP for ranking the factors affecting the successful development of e-HRM. Faliagka et al. (2012) presented a new method for recruiting and ranking job applicants in online recruitment systems using AHP and personality mining approach. Farsijani and Aref Nejad (2011) using AHP method ranked the factors influencing implement of e-HRM.

#### 4.2.8 Green HRM

Decision making on GRHM practices is very important (Leidner et al., 2019). Therefore, decision making techniques such as AHP can be useful in this field. For example, Alavi and Aghakhani (2023) prioritized GHRM practices using the fuzzy analytic hierarchy process. Mehrajunnisa et al. (2022) proposed an AHP framework that can be used to conceptualize and prioritize GHRM practices, supporting green decision-making and the transition to sustainable green development. Goel et al. (2022) identified the most important challenges affecting the adoption of green human resource management using AHP approach. Thakur and Mangla (2019) identified key drivers of sustainable operations management based on human-operational-technological aspects supported by literature and expert opinion. This study proposes that FAHP and Evaluation laboratory methods can be used to prioritize the factors and assess cause and effect relationships between factors.

#### 4.2.9 Knowledge management

Muniz et al. (2022) used AHP for ranking the workers and managers judgments about factors that facilitate knowledge-sharing. Sani et al. (2019) applied fuzzy AHP and fuzzy TOPSIS techniques for knowledge management adoption to financial institutions. AlShamsi and Ajmal (2018) used AHP method to prioritize the critical factors for Knowledge sharing in technology-intensive organizations. Castrogiovanni et al. (2016) applied AHP method to determine which sources of knowledge have the greatest effect on financial entities’ knowledge acquisition and management.

#### 4.2.10 Other applications

In addition to the aforementioned studies, AHP, FAHP, and ANP have also been used in other HRM practices. According to Panazan et al. (2023), the COVID-19 pandemic has caused an unexpected need for change within organizations, especially in terms of human resources management. In this regard, they proposed an integrated ANP-TOPSIS method for ordering preference according to the ideal solution framework. Joshi et al. (2017) applied the AHP method to qualitatively analyze recent trends in human resource management. Abdullah and Zulkifli (2015) evaluated the criteria and dimensions of HRM problem using fuzzy AHP and fuzzy DEMATEL. Chou et al. (2012) applied an integrated fuzzy AHP and fuzzy DEMATEL method to evaluate human resource criteria for science and technology. They first used the AHP approach to evaluate the weight for each criterion and then used DEMATEL to establish the contextual relationships between the criteria. Lin and Hsu (2010) provided an integrated group decision support system (GDSS) to select the appropriate HR capabilities. Their proposed GDSS developed based on the different mathematical and analytical methods such as analytic hierarchy process based on genetic algorithms (GA-AHP), fuzzy mathematics programming, fuzzy set theory, similarity measures, gap analysis, synergy analysis, value chain, HR scorecard, and electronic focus groups. Wu et al. (2010) used a combination of analytic hierarchy process and decision-making trial and evaluation laboratory method to evaluate the criteria of the employment service outreach program personnel. Lin et al. (2009) proposed an evaluation model based on a FAHP approach to rank the factors influencing knowledge sharing. First, they identified 16 features related to four dimensions influencing knowledge sharing. Then, they used FAHP to determine the relative weights concerning these four dimensions and 16 features, and Tseng and Lee (2009) using an AHP/DEA method compared the impact of human resource practices on organizational performance. They used five human resource practices variables (compensation, hiring, training, participation, and motivation) and seven organizational performance variables (employee relations, innovation, employee performance, perceived market performance, corporate financial, productivity, and turnover). Kwak et al. (1997) developed a Human Resource Planning (HRP) model using AHP and Delphi method.

## 5 Discussion and conclusions

**Figure 8:**
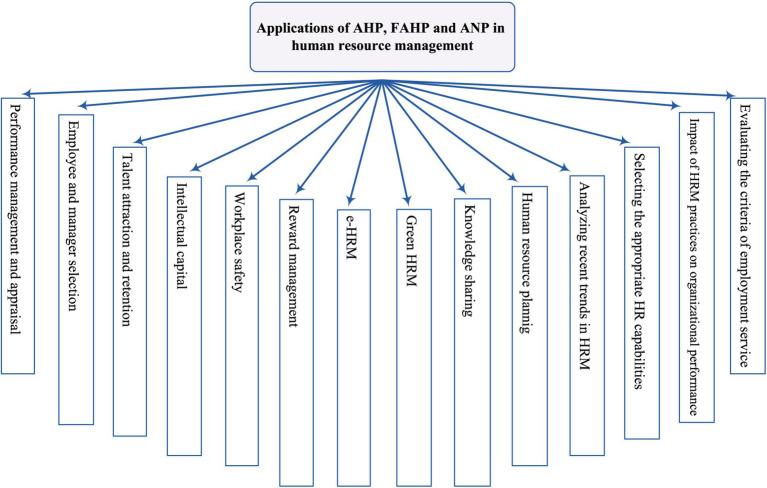
Applications of AHP, FAHP, and ANP in human resource management.

The analytic hierarchy process method has been successfully applied in many fields (Vaidya and Kumar, 2006; Subramanian and Ramanathan, 2012; Bakir and Atalik, 2021). This systematic literature review explored the applications of analytic hierarchy process, fuzzy analytic hierarchy process, and analytic network process in human resource management. By analyzing 180 research articles identified through Scopus and Web of Science, the study revealed a wide range of HRM domains where these methods have been successfully implemented (see Figure 8).

As shown, these domains include performance management, selecting human resources, intellectual capital, workplace safety, green HRM, e-HRM, and more. This research contributes to the existing body of knowledge by providing a comprehensive overview of AHP, FAHP, and ANP applications in HRM.

### 5.1 Managerial implications

The results of current research offer a data-driven approach for various HRM domains, leading to more objective, efficient, and effective HR practices. This study suggests that AHP, FAHP and ANP can significantly improve performance management practices. Managers seek employees who contribute to the organization’s goals and excel in their roles. Performance management is a set of activities designed to achieve the results that companies expect from their employees (Mathis and Jackson, 2008; Andrés et al., 2010). The importance of employee evaluation and its relationship to the organizational outcomes is well documented in different studies (Lowe, 1986; Chang and Hahn, 2006; Ali and Opatha, 2008). Since many organizations do not have an effective method for evaluating employee performance, it is very important to develop a systematic approach to conduct the performance evaluation process at the planning stage (Ahmed et al., 2013). Usually, performance evaluation is done under uncertainty, based on different tactics and strategies and several factors of qualitative and quantitative nature (Manoharan et al., 2011). Common performance evaluation methods such as individual ranking, group ranking, and graphic rating scale do not take into account imprecision and uncertainty of factors (Manoharan et al., 2009). Therefore, fuzzy models can facilitate the decision process of employee evaluation (Golec and Kahya, 2007). Employee evaluation criteria can be both objective and subjective. Therefore, employee evaluation is imprecise, uncertain, and vague, and it is not easy to evaluate its criteria impartially. Fuzzy evaluation eliminates the factors that negatively affect unbiased assessment and promotes fair employee evaluation (Macwan and Sajja, 2012). By adopting AHP, FAHP and ANP, organizations can move toward a more comprehensive, objective, and fair approach to performance management that ultimately fosters a more skilled and engaged workforce.

The topic of employee selection has attracted great attention from both practitioners and researchers for many years (Robertson and Smith, 2001; Breaugh, 2009). Since selecting the right candidate is crucial for employee performance, AHP offer valuable tools for human resource managers. This method can help move beyond traditional selection methods that rely on subjective criteria. Most employee selection methods include a set of relevant criteria with subjective and complex characteristics (Reyes et al., 2003). Therefore, AHP and FAHP will be very useful in the selection process. It can be used, for example, to select creative employees (Hunter et al., 2012). These methods can be integrated with existing selection processes by incorporating the weighting and prioritization derived from AHP/FAHP analysis. Ultimately, AHP/FAHP can empower HR professionals to make more informed selection decisions, leading to a more qualified and effective workforce.

In today’s competitive talent market, attracting and retaining top performers is critical for organizational success (Cable and Turban, 2001; Mathis and Jackson, 2008; Ryan and Delany, 2010). AHP, FAHP and ANP empower HR professionals to make data-driven decisions throughout the talent management process. These methods go beyond traditional selection techniques by enabling the evaluation of both objective and subjective factors influencing talent acquisition and retention. By using AHP to prioritize these factors and establish a robust talent evaluation system, HR can attract high-potential candidates and implement effective retention strategies, leading to a more competitive and successful workforce (Luo and Zhu, 2023).

Based on research findings, AHP, FAHP and ANP offer valuable tools for human resource managers to measure and manage intellectual capital, a key organizational asset (Armstrong, 2009; Safari et al., 2015). These methods can be applied to both human capital and organizational capital. For human capital, AHP can help assess the importance of various employee skills, knowledge, and abilities (Mathis and Jackson, 2008; Martin, 2010) for achieving organizational goals. Similarly, AHP can be used to prioritize and rank measurement indicators of organizational capital. By providing a structured framework for evaluating both human and organizational capital, AHP empowers HR to make informed decisions that maximize the value of these intangible assets.

Safety management is an important element in the workplace (Dagdeviren and Yuksel, 2008). The primary goals of effective safety programs are to protect the physical well-being of employees and prevent work-related accidents and injuries in the organizations. The growing problem of risk management and violence in the workplace has led to increased attention to the issue of safety and security of employees (Mathis and Jackson, 2008). FAHP provides a valuable tool for safety managers by enabling the simultaneous evaluation of multiple factors influencing workplace safety. They can prioritize areas for improvement and allocate resources more effectively, ultimately leading to a safer work environment for employees.

Traditionally, reward management focused solely on financial incentives. AHP, FAHP and ANP offer a more nuanced approach by enabling HR to evaluate both monetary and non-monetary rewards (Mathis and Jackson, 2008; Martin, 2010), considering their relative importance in attracting, retaining, and motivating employees. By using AHP to assess the value proposition of different reward options, HR can design more effective compensation packages that address the diverse needs and priorities of today’s workforce, leading to increased employee satisfaction and organizational success.

In recent years there is a great interest to research in the field of e-HRM (Strohmeier, 2007; Stone and Lukaszewski, 2009; Schalk et al., 2013; Nyathi and Kekwaletswe, 2023) and many studies have been conducted in various fields of e-HRM such as selection (Chapman and Webster, 2003), performance management (Cardy and Miller, 2005), recruitment (Stone et al., 2003), and payroll administration (Teo et al., 2001). AHP empowers HR professionals to optimize the effectiveness of e-HRM systems. By using AHP, HR can ensure successful implementation of e-HRM initiatives, maximizing the return on investment in digital HR technologies.

Green human resources management is the integration of traditional human resource practices such as rules, procedures, policies and strategies with the latest green and environmentally sustainable practices (Goel et al., 2022). Through the use of HR philosophies, policies and practices, GHRM promotes the sustainable use of resources and prevents harm arising from environmental concerns in business organizations (Zoogah, 2011). As organizations strive for environmental sustainability, AHP, FAHP and ANP emerge as valuable tools for implementing effective green HRM practices. These methods can guide decision-making by enabling HR professionals to prioritize different GHRM initiatives, such as green training programs or eco-friendly recruitment practices. By incorporating these methods, HR can contribute to a more environmentally conscious organizational culture and support the transition toward a greener future.

Although knowledge management cannot be directly considered as one of the functions of human resource management; however, there is a very close relationship between HRM practices and knowledge management (Hislop, 2003; Edvardsson, 2008; Gope et al., 2018). For example, it is increasingly acknowledged that the success of knowledge management initiatives fundamentally depends on the presence of employees willing to share their knowledge, and HRM frameworks and concepts can be used to enhance our understanding of the factors that determine workers’ willingness (or reluctance) to share knowledge. AHP/FAHP methods can be used to rank factors influencing employee willingness to share knowledge, such as trust or incentives. By using AHP/FAHP, HR can prioritize initiatives that promote knowledge sharing within the organization. This approach empowers HR to create a more knowledge-sharing friendly environment, fostering innovation and organizational learning.

### 5.2 Limitations and future research directions

While our systematic review provided an overview of the applications of AHP in HRM, our approach has certain limitations. Research published in the database or in languages other than those selected should be considered by future studies. In addition, unpublished studies and gray literature may be considered by other researchers. As the results showed, AHP, FAHP and ANP approaches have had the most applications in performance management and appraisal as well as selecting human resources. Therefore, additional research is needed in various areas of human resource management. In addition, reviewing the applications of other fuzzy set theories in HRM (Dursun and Karsak, 2010; Boran et al., 2011; Kelemenis et al., 2011) can be considered in future studies. Furthermore, the AHP method can also be used in the field of organizational behavior management. This study focused on AHP, FAHP, and ANP as quantitative decision-making methods in HRM. While these methods offer valuable insights, they may not fully capture the complexities of HRM decisions. Assigning objective weights to criteria can be challenging due to the subjective nature of HRM. Additionally, these methods might struggle to fully account for factors like employee motivation, organizational culture, and soft skills, which can be crucial considerations in HRM. Future research could explore how quantitative methods can be integrated with qualitative approaches. This combined approach could provide a more comprehensive understanding of HRM decision-making processes. Additionally, future studies could investigate the use of qualitative methods in specific HRM areas, such as recruitment, performance management, or talent retention.

## Author contributions

RS: Conceptualization, Supervision, Writing – original draft, Writing – review & editing. MZ: Methodology, Writing – original draft.
